# Mutations in the *TOPORS* gene cause 1% of autosomal dominant retinitis pigmentosa

**Published:** 2008-05-19

**Authors:** Sara J. Bowne, Lori. S. Sullivan, Anisa I. Gire, David G. Birch, Dianna Hughbanks-Wheaton, John R. Heckenlively, Stephen P. Daiger

**Affiliations:** 1 Human Genetics Center, School of Public Health, Houston, TX; 2 Retina Foundation of the Southwest, Dallas, TX; 3 Kellogg Eye Center, University of Michigan, Ann Arbor, MI; 4 Department of Ophthalmology and Visual Science, The University of Texas Health Science Center at Houston, Houston, TX

## Abstract

**Purpose:**

The purpose of this project was to determine if mutations, including large insertions or deletions, in the recently identified RP31 gene *topoisomerase I-binding arginine-serine rich (RS)* protein (*TOPORS*), cause an appreciable fraction of autosomal dominant retinitis pigmentosa (adRP).

**Methods:**

An adRP cohort of 215 families was used to determine the frequency of *TOPORS* mutations. We looked for mutations in *TOPORS* by testing 89 probands from the cohort without mutations in other known adRP genes. Mutation detection was performed by fluorescent capillary sequencing and by multiplex ligation probe amplification.

**Results:**

Two different *TOPORS* mutations, p.Glu808X and p.Arg857GlyfsX9, were each identified in one proband. Patients with these mutations exhibited clinical signs typical of advanced adRP. No large deletions or insertions of *TOPORS* were identified in our study.

**Conclusions:**

Point mutations and small insertions or deletions in *TOPORS* cause approximately 1% of adRP. Large deletions or insertions of *TOPORS* are not an appreciable cause of adRP. Contrary to previous reports, no distinct clinical phenotype was seen in these patients.

## Introduction

Retinitis pigmentosa (RP) is a heterogeneous form of inherited blindness initially characterized by night blindness and peripheral vision loss, usually culminating in legal or complete blindness. Approximately one out of 3,000–4,000 individuals is affected with RP [1]. RP can be inherited in an autosomal dominant (adRP), autosomal recessive (arRP), or X-linked (XlRP) pattern and to date, 16 autosomal dominant, 18 autosomal recessive, and six X-linked loci have been identified, with some genes causing multiple overlapping diseases. (RetNet) [2].

Disease-associated genes have been identified for 15 of the 16 adRP loci (RetNet) [3]. The most recent disease-associated gene identified was for the RP31 form of adRP [4]. Mutations in the *topoisomerase I-binding arginine-serine rich (RS) gene* (*TOPORS*) cause RP31 [5]. Initial findings identified two different *TOPORS* mutations associated with disease, both of which were small insertions/deletions resulting in frame-shifts and premature termination of the protein. The mutant TOPORS protein was not detected in lymphoblast cell lines from patients with these mutations.

Examination of additional adRP probands for mutations in *TOPORS* will provide information on mutation frequency and may potentially provide additional biologic information regarding the disease mechanism. Since haploinsufficiency is believed to be the likely disease mechanism associated with *TOPORS* mutations, it is possible that large deletions, or copy number variants (CNV) in *TOPORS*, may also be a cause of adRP. This project examines a well defined adRP cohort using both standard sequencing methodology and multiplex ligation probe amplification (MLPA) to look for CNVs [6,7].

## Methods

### Autosomal dominant retinitis pigmentosa cohort and controls

The cohort of 215 adRP probands used in this study has been described in detail previously [6-8]. This cohort is a set of 215 families which, based on pedigree analyses, have a high likelihood of having adRP. Each proband has been previously tested for mutations in the complete coding region of *CA4*, *CRX*, *FSCN2*, *IMPDH1*, *NRL*, *PRPF31*, *RDS*, *RHO*, *ROM1*, and *RP9*. Samples were also screened for mutations in mutational “hot spots” of *RP1*, *PRPF3*, *PRPF8*, and *NR2E3*. Likely disease-causing mutations have been identified in 126 of the 215 families. Probands from the remaining 89 families were tested in this study. A set of 90 unrelated normal control samples obtained from the Centre dEtude du Polymorphisme Humain were also tested for the presence of the two mutations identified in the cohort samples [9].

This study was performed in accordance with the Declaration of Helsinki, and informed consent was obtained from all participants. This research was approved by the Committee for the Protection of Human Subjects at the University of Texas Health Science Center at Houston and by the respective human subjects’ review boards at each participating institution.

### Mutation detection

#### Sequencing analyses

PCR product sequencing was employed to screen patient DNA for mutations in the entire coding region and flanking intron/exon junction of *TOPORS*. PCR amplification and sequencing were performed as previously described using the primers in Table 1 [6]. Briefly, exons 1 and 2 were each amplified using 30–50 ng of genomic DNA and AmpliTaq Gold (Applied Biosystems, Foster City, CA) in a 12.5 μl reaction for 35 cycles. Exon 3 was amplified in two pieces using 100 ng of genomic DNA and either AmpliTaq Gold or HotStarTaq DNA polymerase (Qiagen, Valencia, CA) with Q-solution, in a 25 μl reaction for 35–40 cycles.

**Table 1:** Primers used in polymerase chain reaction amplification and sequencing

Exon	Amplification primers (5’-3’)	Annealing temperature	Sequencing primers (5’-3’)
1	ACGTAAGAAGCGGAAGATCG	63 °C	Same as amplification
	GCCTGGGAGGTTACTGTAAGG		
2	GTGGGTCTC GCT CTC TGC	63 °C	Same as amplification
	CCCATTGTTCCGAATCTCAC		
3A	TCAAGGTCTTTATTTGCATTTTTG	52 °C	TCAAGGTCTTTATTTGCATTTTTG
	GCTTCTTCTGGACCAACTGC		AGAACAACAACTCCACCG
			GCCTTCACAGATTAGTCCC
			GAGAAACGATCTACATCATTGTC
			AGTTGGCCTCCTTACTGCAA
			GACCACTCCTGTACACAGCGAAAAC
			TTCTGGGGTCCTCTCAGCTA
			GGCTTCTTCTGGACCAACTGC
3B	TAGCTGAGAGGACCCCAGAA	58 °C	AGTTGGTCCAGAAGAAGCCA
	GGAGGAAGAGAGTTTTCACCAA		TACAAAACACGGCATTTGGA
			AAGACCCGGAGCCTAAGTGT
			GATGAAGATTTTTGGTAATGACTG

The above PCR and sequencing primers were used to identify *TOPORS* mutations in our cohort of adRP patients. For exons 1 and 2, the same primers were used for amplification and sequencing reactions. For exons 3A and 3B, several nested sequencing primers were used to span each amplified PCR product.

PCR products were treated with ExoSapIt (USB, Cleveland, OH) and sequenced unidirectionally with BigDye v1.1 (Applied Biosystems) and the primers described in Table 1. Sequence reactions were purified using BigDye® Xterminator Kit (Applied Biosystems) and the manufacturer’s protocol. Purified reactions were run on an ABI 3100-Avant Genetic Analyzer (Applied Biosystems) and analyzed using SeqScape Software (Applied Biosystems).

#### Multiplex ligation probe amplification analyses

MLPA analyses were performed as previously described using eight probe pairs designed to span each of the *TOPORS* amplification primers (Table 2), seven control probe pairs, and the EK1 kit (MRC-Holland, Amsterdam, The Netherlands) [7]. Briefly, probes were selected based on the recommendations of MRC-Holland and Raw-Probe Software (MRC-Holland). All half probes were synthesized by Sigma Genosys (The Woodlands, TX) and desalted at the time of synthesis.

**Table 2:** Probes used in multiplex ligation probe amplification

Probe set	Probe location*	5′ half probe (5′-3′)	Phosphorylated 3′ half probe (5′-3′)
Amp1 Front	32542621–32542674	GGGTTCCCTAAGGGTTGGA TGGCGGGTACACCCAGC AGCCCTTAC	pGTAAGAAGCGGAAGATCGTATCCT CCAGTCTAGATTGGATCTTGCTGG CAC
Amp 1 Back	32542362–32542421	GGGTTCCCTAAGGGTTGGA GGCAGCAGTCCGCGGGA GCTGGCGGGAG	pCTGCGGGCCTTACAGTAACCTCCC AGGCGGTGTCTAGATTGGATCTTGCTGGCAC
Amp 2 Front	32540968–32541035	GGGTTCCCTAAGGGTTGGA CTGGGGGGTCTCGCTCTC TGCCCTGCTTCCGAG	pCTGCCATTGGTGATGAGCCCTTTG CGTCACATCTAGATTGGATCTTGC TGGCAC
Amp 2 Back	32540731–32540800	GGGTTCCCTAAGGGTTGGA CAGCCAGGCCTGCGCCG GCATCCTCCGAG	pGTGAGTGAGATTCGGAACAAT GGG ACGCGGGGGTCGGAAGGTCTAGATTGGATCTTGCTGGCAC
Amp 3A Front	32534380–32534475	GGGTTCCCTAAGGGTTGGA CAGTGCCCCTTTATAAA ATAAAACAAAAGTAATGG GTCACTTAAGTATTTTCAC	pCAAAATAAGTTTCAAGGTCTTTAT TTGCATTTTTGTTGAGACTCTAGATTGGATCTTGCTGGCAC
Amp 3A Back	32532520–32532601	GGGTTCCCTAAGGGTTGGA GAGACAAAAAGAGATCA AGAACTAGAGATAGCAG TTGGTC	pCAGAAGAAGCCAAACTCTGTCTCT AAGTAGTGAAAGCACAAGTCTAGATTGGATCTTGCTGGCAC
Amp 3B Front	32533017–32533090	GGGTTCCCTAAGGGTTGGA GTGTCATTGTTGGGTTTGTT AAACCACTAGCTGAGAG	pGACCCCAGAACTTGTTGAACTGTC CTCTGATTCTGAGTCTAGATTGGATCTTGCTGGCAC
Amp 3B Back	32531257–32531342	GGGTTCCCTAAGGGTTGGA GGAAAAAGGAAGAATGT CGTCTACTGCAGTCTATT TAAAGAT	pGACATTTGGTGAAAACTCTCTTCC TCCTTACAATATTTTAAATGTCTAGATTGGATCTTGCTGGCAC

Half probes were designed to anneal to the equivalent genomic sequence as the sequencing amplification primers described in Table 1. Each probe sequence contains a universal half probe sequence which is underlined. The asterisk indicates the position on chromosome 9 based on the University of California Santa Cruz human genome assembly of March 2006 (hg18).

Probe cocktails were hybridized overnight with 25–50 ng of genomic DNA, ligated, and then PCR amplified according to the DNA detection-quantification protocol recommended by MRC-Holland. PCR product was diluted in deionized formamide (Applied Biosystems) containing GeneScan-500 LIZ size standards (Applied Biosystems) and run on a 3100-Avant Genetic Analyzer. Dosage quotients (DQs) were calculated for each *TOPORS* probe as described by Stern et al. using GeneMapper (Applied Biosystems) and Excel (Microsoft, Redmond, WA) software [10]. A DQ of 1.0 indicated the presence of two alleles while a 0.5 or 1.5 suggested that either a deletion or duplication of the target sequence, respectively.

## Results

### Sequencing analyses

We tested genomic DNA from 89 adRP probands for mutations in *TOPORS* using fluorescent capillary sequencing. This procedure detected likely disease-causing mutations in two of the probands tested. Each of these mutations was heterozygous, consistent with autosomal dominant inheritance. A 1 bp deletion at nucleotide 2,569 was detected in the first proband from family UTAD102 (c.2569delA; p.Arg857GlyfsX9). This deletion caused a frame-shift at amino acid residue 857, and was predicted to result in the addition of eight incorrect amino acids followed by premature termination. Unfortunately, no additional family members were available for testing. Analysis of 180 chromosomes from normal controls failed to find this DNA change.

**Figure 1:**
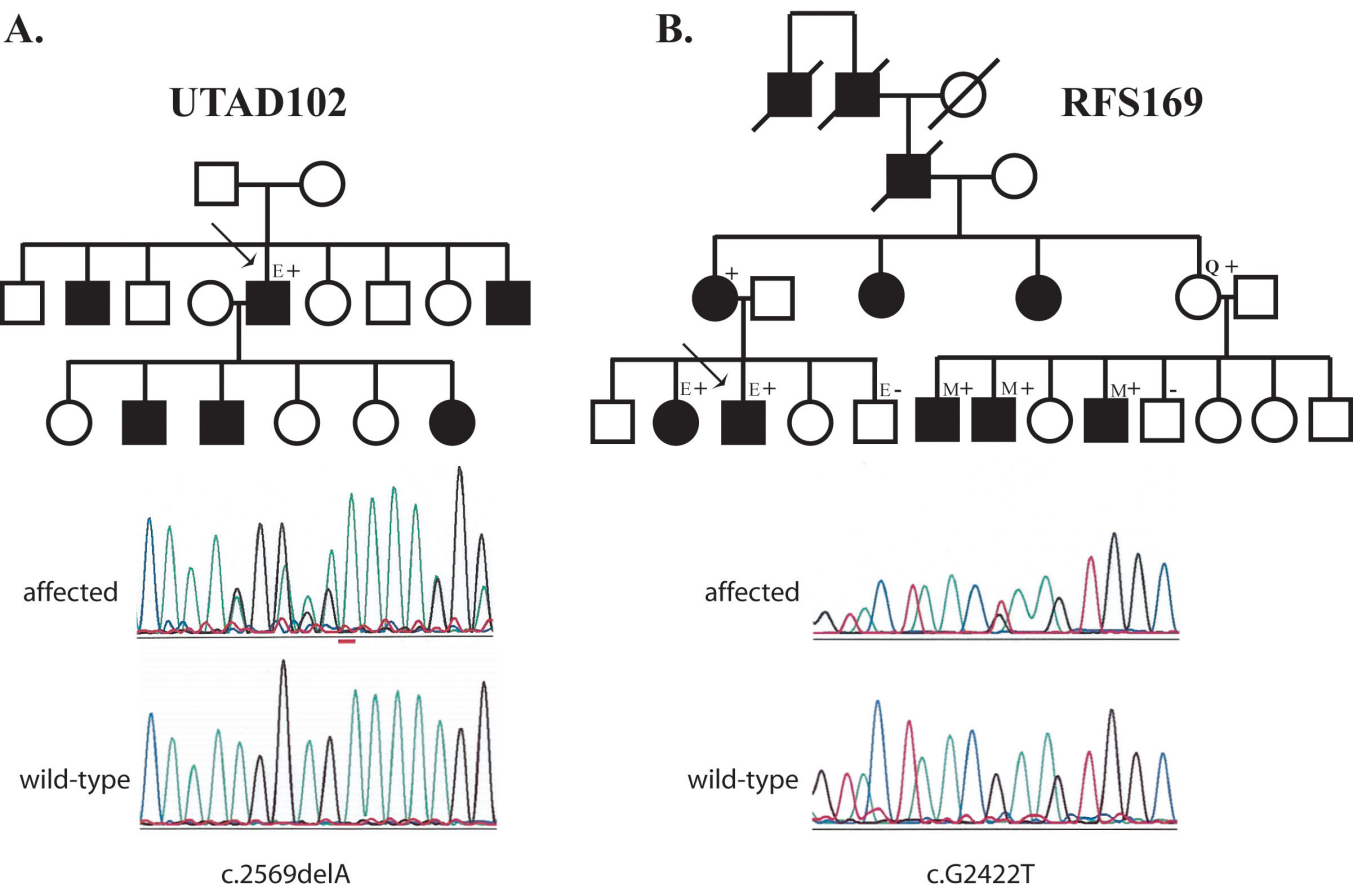
Pedigrees of families with TOPORS mutations. **A:** This family has the p.Arg857GlyfsX9 (c.2569delA) mutation. **B:** RFS169. This family has the p.Glu808X (c.2422C>T) mutation. Circles indicate females; squares indicate males. Black filled symbols are affected individuals, open symbols are unaffected individuals, and the “Q” indicated an individual in New York who reports being asymptomatic. “E”s indicate individuals who had eye examination at either the Retina Foundation of the Southwest or the Jules Stein Eye Institute. “M”s indicate individuals for whom ophthalmic medical records were reviewed. Plus signs show individuals whose DNA tested positive for the family's mutation; minus signs are individuals whose DNA tested negative for the family's mutations.

The second *TOPORS* mutation was a c.G2422T nonsense change, which resulted in a p.Glu808X in family RFS169. Analysis of this mutation in eight additional family members demonstrated that this mutation tracks with disease (Figure 1). This mutation was also not found in the 180 normal chromosomes tested. Both of the mutations identified in this study, like those identified previously by Chakarova and colleagues [5], resulted in premature termination of the TOPORS protein.

During our analyses we also identified two missense changes not found in database of single nucleotide polymorphisms (dbSNP). The first change, a p.Pro20Ser (c.C58T), was found in four apparently unrelated Caucasian probands. Additional family members from one family were tested for the presence of the variant. The p.Pro20Ser variant was not found in four affected individuals or obligate carriers in this family and hence was not considered disease-causing. The second variant, p.Thr782Ala (c.A2344G), was found in two Caucasian probands. Testing of additional family members showed that this variant also did not track with disease and therefore, is benign.

### Multiplex ligation probe amplification

Work recently published by Chakarova et al. [5] suggested that haploinsufficiency is the disease mechanism of *TOPORS* mutations. This is based on protein analysis of the two previously identified *TOPORS* mutations that, like the ones found in this study, result in a premature protein termination. The protein analyses of Chakarova et al. [5] failed to detect the mutant protein in lymphoblast cell lines from either mutation.

Given that haploinsufficiency is a likely disease mechanism for *TOPORS* mutations, it is possible that a gross deletion or CNV of *TOPORS* would also lead to retinal disease. To determine if CNVs are a common cause of RP, we performed MLPA analyses of *TOPORS* using a series of eight custom probe sets designed in our laboratory. These probes were designed to overlap with the original amplification primers used in sequence analysis such that any failure to amplify would also likely be detected. MLPA analyses of *TOPORS* in the 89 individuals from our adRP cohort did not detect CNVs.

### Clinical description

#### UTAD102

The prospectus was a 52-year-old Hispanic male with only hand motion vision in both eyes. He had two brothers and three children diagnosed with RP (Figure 1A). Extensive bone-spicule-like pigment deposits and severely attenuated blood vessels were seen bilaterally, consistent with end stage RP. Optic disc pallor and macular retinal pigment epithelium atrophy were also present. Goldman visual fields were not possible due to the patient’s poor vision.

#### RFS169

The prospectus was a 31-year-old Caucasian male whose major complaint was difficulty going down steps and curbs in dim light or at night. He was aware that his peripheral vision was poor and reported frequent inability to locate objects. His sister, mother, maternal aunt, maternal grandfather, maternal great-grandfather, and maternal great uncle had been diagnosed with RP (Figure 1B). He reported that his mother had severe night blindness at age 20 and was now 60 years old with extremely poor vision.

Visual acuity was 20/20–2 OD and 20/20–2 OS. Slit lamp findings were normal bilaterally. Ophthalmoscopy revealed clear vitreous ocular uterque (OU) and normal discs. Both maculas were normal without edema. The midperiphery contained numerous bone-spicule-like pigment deposits. The retinal arterioles were slightly narrowed by comparison to the veins. Static perimetry was obtained with a Humphrey Field Analyzer (Humphrey Instruments, San Leandro, CA), using programs 30–2 and 60–2. Sensitivity was minimally disturbed in the fovea and central 15 °. Sensitivity was zero at most locations beyond 7.5 ° eccentricity. The 60–2 field showed a region of preserved function in the lower temporal field.

Visual thresholds following 45 min of dark adaptation were elevated by 1.5 log unit. Full-field electroretinograms (ERGs) showed that the International Society for Clinical Electrophysiology of Vision (ISCEV)-standard rod response was not detectable. The maximum rod photoresponse to a 4.2 log scot td-sec flash was 10 μV, compared to a lower limit of normal of 155 μV. Cone b-wave amplitude to 31 Hz flicker was 6.4 μV, compared to a lower limit of normal of 35 μV. Cone b-wave implicit time was delayed by 9.3 msec. The maximum cone photoresponse obtained in the presence of a 3.2 log td background was 7.7 μV, compared to a lower limit of normal of 33.7 μV.

**Figure 2:**
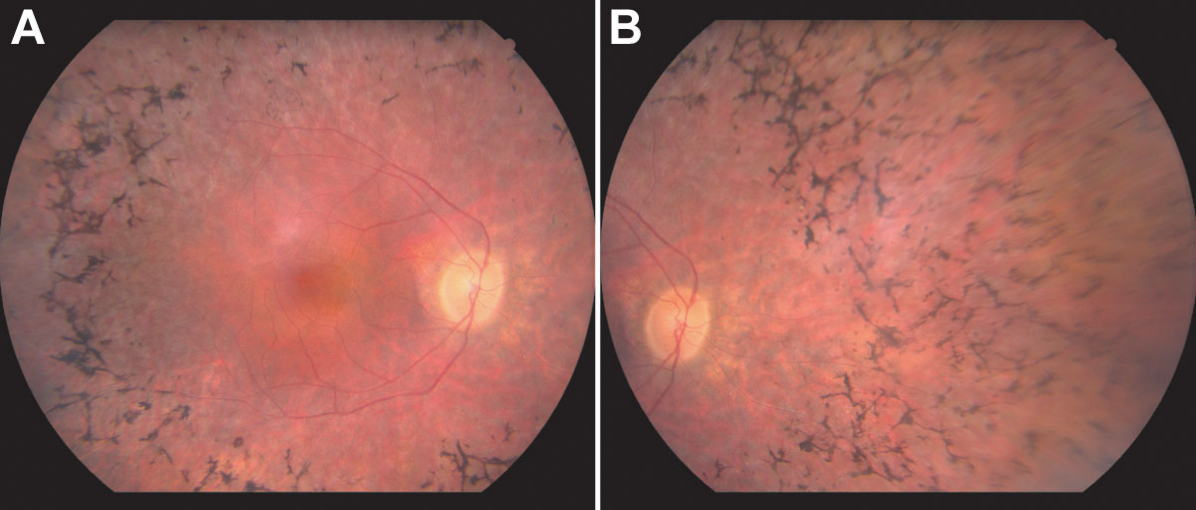
Fundus photographs from RFS169. Right midpheripheral fundus (**A**) and right peripheral fundus (**B)** photographs from the 41-year-old sister of the proband from family RFS169. The midperiphery of both eyes contained numerous bone-spicule-like pigment deposits and the retinal arterioles were slightly narrowed by comparison to the veins. There was no evidence of a perivascular cuff of retinal pigment epithelium atrophy around the superior and inferior arcades in this family.

The sister of the prospectus was 41 years old at the time of examination. She too was aware of night vision and side vision impairment. In addition, she complained of poor central vision. Ophthalmoscopy revealed clear vitreous OU and normal discs. Both maculae were normal without edema. The midperiphery contained numerous bone-spicule-like pigment deposits. The retinal arterioles were slightly narrowed by comparison to the veins (Figure 2).

Visual acuity was 20/20–2 OD and 20/20–2 OS. Humphrey perimetric values could only be obtained from the central four locations on the 30–2 field; all locations were decreased in sensitivity by at least 20 dB with the exception of the fovea, where sensitivity was within the normal range. Full-field ERGs showed that the ISCEV-standard rod response was not detectable, as was the maximum rod photoresponse. Cone b-wave amplitude to 31 Hz flicker was 0.5 μV, compared to a lower limit of normal of 35 μV. Cone b-wave implicit time was within the normal range.

Medical record examination also provided some detail regarding the clinical details of the nuclear family found at the right of the pedigree (Figure 1B). The oldest affected male cousin of the prospectus was last examined at age 42. At that time his visual acuity OU was 20/20. He was reported to have severe field constriction but no fields were available. Fundus drawings indicated crescent-like areas of atrophy around the arcades. The younger brother of this individual was last examined when he was 30 years old. His acuity was 20/30 in each eye. Humphrey fields measured less than 10 degrees in each eye, and standard ERGs (no computer averaging) were not detectable. Fundus appearance was typical for RP; specifically there was no note of atrophy around the arcade vessels.

The youngest affected brother in this nuclear family was last examined at age 32. His visual acuity was 20/150 in each eye. He had a long history of keratoconus with corneal grafts in each eye. Due to these corneal problems, there was no fundus or visual field information available.

Medical records were not immediately available from the mother of these three brothers. Results from a self-reported questionnaire, completed at age 66, indicated that she did not have any trouble with her vision other than glasses needed for acuity correction. Specifically, she reported no trouble seeing at night or with her peripheral vision. This suggests that she is an asymptomatic carrier of the disease-causing mutation found in her three affected sons.

## Discussion

Based on our analyses, mutations in *TOPORS* cause approximately 1% of adRP. Further, these mutations are most likely to be nonsense changes or small insertion/deletions that lead to premature termination of the protein [5]. No CNVs were identified in this study, making it unlikely that *TOPORS* CNVs cause an appreciable fraction of adRP. Since our adRP cohort is composed primarily of probands of Western European origin, it is possible that the *TOPORS* mutation frequency, like other adRP gene mutation frequencies, could be different in other populations [3]. Additional studies will be needed to address this question.

Chakarova et al. [5] reported finding a unique clinical phenotype in the large family that originally mapped the RP31 locus. In four children they observed a perivascular cuff of retinal pigment epithelium atrophy in the superior and inferior arcades that progressed into pigmentary retinopathy with choroidal sclerosis. The unique perivascular cuff was not seen in any of our examined patients, but its absence could easily be due to the later age at which our patients were examined. Several members of the original RP31 family were also reported to be asymptomatic despite carrying the *TOPORS* mutation [4]. One member from the RFS169 family discussed in this study is known to carry the mutation and has reported being asymptomatic (Figure 1B).

**Figure 3:**
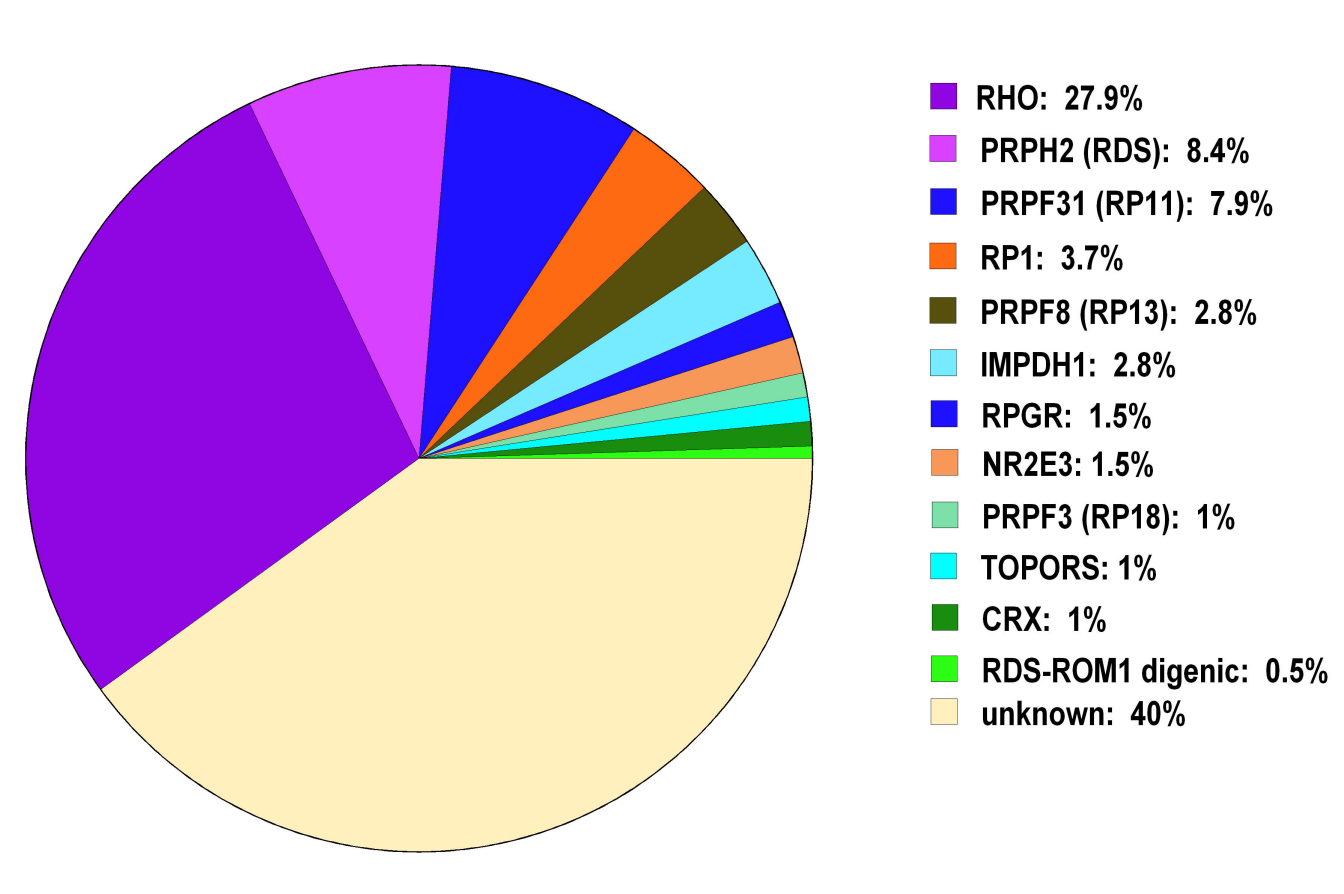
Frequency of autosomal dominant retinitis pigmentosa mutations found in the autosomal dominant retinitis pigmentosa cohort by gene. Gene abbreviations: rhodopsin (RHO); peripherin 2 (PRPH2); pre-mRNA processing factor 31 homolog (PRPF31); retinitis pigmentosa 1 (RP1); pre-mRNA processing factor 8 homolog (PRPF8); inosine monophosphate dehydrogenase 1 (IMPDH1); retinitis pigmentosa GTPase regulator (RPGR); nuclear receptor subfamily 2, group E, member 3 (NR2E3); pre-mRNA processing factor 3 homolog (PRPF3); topoisomerase I-binding arginine-serine rich gene (TOPORS); cone-rod otx-like photoreceptor homeobox transcription factor (CRX); retinal outer segment membrane protein 1 (ROM1). Testing identified mutations in 60% of our autosomal dominant retinitis pigmentosa cohort of 215 families. Mutations have yet to be identified in the remaining 40%.

With the addition of *TOPORS* to the list of adRP-asssociated genes, mutations can now be identified in 60% of individuals with adRP (Figure 3) [6-8]. Mutations in the remaining 40% of affected individuals remain to be identified. It is clear that there are still additional adRP genes to be identified.
